# Genotype-phenotype correlations in children and adolescents with nonclassical congenital adrenal hyperplasia due to 21-hydroxylase deficiency

**DOI:** 10.1186/s40348-020-00100-w

**Published:** 2020-07-09

**Authors:** Helmuth-Günther Dörr, Nadja Schulze, Markus Bettendorf, Gerhard Binder, Walter Bonfig, Christian Denzer, Desiree Dunstheimer, Kirsten Salzgeber, Heinrich Schmidt, Karl Otfried Schwab, Egbert Voss, Martin Wabitsch, Joachim Wölfle

**Affiliations:** 1 Paediatric Endocrinology, University Children’s Hospital, Erlangen, Germany; 2 Paediatric Endocrinology, University Children’s Hospital, Heidelberg, Germany; 3 Paediatric Endocrinology, University Children’s Hospital, Tübingen, Germany; 4 Departement of Paediatrics, Hospital Wels-Grieskirchen, Wels, Austria; 5 Paediatric Endocrinology, University Children’s Hospital, Ulm, Germany; 6 University Children’s Hospital I, Augsburg, Germany; 7 Medical Office, Endokrinologikum, Ulm, Germany; 8 Paediatric Endocrinology, University Children’s Hospital, Munich, Germany; 9 Paediatric Endocrinology, University Children’s Hospital, Freiburg, Germany; 10 Departament of Paediatrics, Cnopfsche Kinderklinik, Nuremberg, Germany

**Keywords:** CYP21A2 mutations, 21-Hydroxylase deficiency, Androgenisation, Premature pubarche, 17OHP, ACTH stimulation test

## Abstract

**Background:**

Nonclassical congenital adrenal hyperplasia due to 21-hydroxylase deficiency is caused by mutations in the active 21-hydroxylase gene (CYP21A2). The clinical symptoms can vary greatly. To date, no systematic studies have been undertaken in Germany.

**Aims:**

Description of the phenotype, evaluation of the diagnostics and genotype-phenotype correlation

**Patients and methodology:**

Retrospective analysis of the data of 134 patients (age range 0.1–18.6 years) in a multicentre study covering 10 paediatric endocrinology centres in Bavaria and Baden-Württemberg. The data was gathered on site from the medical records. Two hundred and thirty-three alleles with a mutation of the CYP21A2 gene were identified in 126 patients. A genotype-phenotype correlation of the mutation findings was undertaken (C1, severe/mild; C2, mild/mild). Individuals with a heterozygous mutation of the CYP21A2 were also included (C3). The data was collected with the approval of the ethics committee of the University Hospital of Erlangen during the period of 2014 and 2015.

**Results (MW ± SD):**

One hundred and seventeen out of 134 patients (115 f, 29 m) were symptomatic. The chronological age (CA) at diagnosis was 7.1 ± 4.4 years. The most frequent symptom (73.5%) was premature pubarche. The height-SDS on diagnosis was 0.8 ± 1.3 and the BMI-SDS was 0.8 ± 1.2. Bone age (BA) was ascertained in 82.9% of the symptomatic patients. The difference between BA and CA was 1.9 ± 1.4 years. Basal 17OHP concentrations were 14.5 ± 19.1 ng/ml (18 patients < 2 ng/ml). In total, 58.1% mild and 34.7% severe mutations were found. The most common mutation was p.Val281Leu (39.1%); 65.8% of the patients could be allocated to group C1. No phenotypical differences were found between the 3 mutation groups. The 17OHP levels (basal and after ACTH) in the standard ACTH stimulation test were highest in group C1 and also significantly higher in group C2 as in C3, the ACTH-stimulated cortisol levels (ng/ml) were significantly lower in groups C1 (192.1 ± 62.5) and C2 (218 ± 50) than in C3 (297.3 ± 98.7).

**Conclusion:**

Most of the patients have symptoms of mild androgenisation. Male patients are underdiagnosed. Diagnostics are not standardised. Differences between the types of mutations are found in the hormone concentrations but not in phenotype. We speculate that further, as yet not clearly defined, factors are responsible for the development of the respective phenotypes.

## Background

Nonclassical congenital adrenal hyperplasia due to 21-hydroxylase deficiency (NC-CAH) is caused by mutations in the active 21-hydroxylase gene (CYP21A2) and is the most common congenital disorder of steroid biosynthesis of the adrenal gland, with an estimated prevalence of 1:200–1:1000 in the Caucasian population [1, 2]. The mutations which result in classic congenital adrenal hyperplasia (CAH) or NC-CAH are classified internationally according to the residual activity of 21-hydroxylase in the mutation groups 0, A, B and C [3–5]. Severe mutations are found in groups 0, A and B while the patients with NC-CAH belong to mutation group C. Most patients are compound heterozygous, i.e. the genotype comprises a combination of mutations, which result in a mild reduction in enzyme activity on one allele and of mutations causing either a total (0% residual activity), a serious (about 2–5% residual activity) or a mild (20–60% residual activity) reduction in enzyme activity on the other alleles [4, 6]. The phenotype is influenced by the activity of the less affected allele, and the residual activity of 21-hydroxylase in the patients is around 20–50% [7]. However, the phenotypical degree of severity within a genotype can vary greatly and different phenotypes can be associated with the same mutation [8].

To date, there are no systematic studies for children with NC-CAH in Germany, and numbers on prevalence are also lacking. At the bi-annual meeting of paediatric endocrinologists in Bavaria and Baden-Württemberg, it was decided to analyse data of affected children in order to answer questions regarding clinical presentation, laboratory and molecular genetic diagnostics and the correlation of genotype to phenotype.

## Patients and methods

The study included 134 children and adolescents (age range 0.1–18.6 years) born between 1972 and 2014 who are treated in 10 different paediatric endocrinology centres in Bavaria and Baden-Württemberg. The study was planned during the bi-annual meeting of paediatric endocrinologists in Ulm. The data was collected retrospectively in the participating hospitals by one person (N.Sch.) in 2014 and 2015, or the data was entered into an Excel table in those centres with few patients.

The height and the body mass index (BMI) of the patients were calculated in standard deviation scores (SDS) in accordance with the reference values of Kromeyer-Hauschild et al. [9]. An SDS value specifies the difference of a measured value to the mean value of the reference population of the same age and is calculated from the difference of the measured value (actual value) and the mean value (nominal value) divided by the corresponding standard deviation of the mean value. Bone age (X-ray of the left hand) was evaluated according to the Greulich and Pyle Atlas method [10]. Pubarche before the 8th birthday in girls and before the 9th birthday in boys was assessed as premature pubarche.

The serum concentrations of 17-hydroxyprogesterone (17-OHP) and cortisol were measured in the individual centres according to the directive of the German Medical Association for the Quality Assurance of Laboratory Medical Examinations (Rili-BÄK). The blood samples were taken in the morning and in the early follicular phase in girls who were menstruating. A standard ACTH stimulation test was carried out on 83 patients (i.v. 250 μg, blood samples taken at 0 and 60 min) and 17-OHP and cortisol were measured. The following are conversion factors for conventional units to SI units: 17OHP, ng/ml × 3 = nmol/l; cortisol, ng/ml × 2.76 = nmol/l.

The molecular genetic diagnostics with complete sequencing of the CYP21A2 gene, and additionally with different methods (MLPA, semi-quantitative PCR) an analysis of the number of copies of the CYP21A2 gene in comparison with the pseudogene CYP21A1P, was performed in different molecular genetic laboratories. Results of the molecular genetic analysis of 126 patients were available. The patients were divided into three groups according to the number of allele combinations of the CYP21A2 gene which were found. Group C1 is compound heterozygous patients with a severe mutation on one allele (mutations from groups 0, A or B) and a mild mutation on the other allele (mutation from group C), group C2 is patients with a mild mutation (heterozygous or homozygous) on both alleles (mutation from group C) and group C3 is heterozygous individuals with only a mild heterozygous mutation on one allele.

### Statistics

All statistical analyses were performed using SPSS 23.0 software (IBM Inc., USA), Quantitative data are presented as the mean ± SD. Normality of the sample was examined by the Shapiro-Wilk test. Changes in clinical and laboratory variables between the different mutation groups were analysed using one-way ANOVA, followed by the post hoc comparisons using Tukey’s test. Statistical significance was considered with a 2-sided *P* value of < 0.05.

## Results

### Clinical findings

The diagnosis was made in 134 patients (105 f, 29 m) at a mean age of 7.1 ± 4.4 (SD) years. Most of the children were diagnosed between the ages of 6 and 10 years (Table 1). 78.4% of patients were female; the ratio of female to male was 3.6:1. One hundred and seventeen patients (97 f, 20 m) presented to outpatients‘ clinics with a variety of clinical symptoms, while 17 (8 f, 7 m) did not yet have any symptoms at the time the data was collected. In these cases, the diagnosis was made in the process of prenatal diagnostics (*n* = 4), in CAH newborn screening (*n* = 6) and during examination of family members with affected siblings (*n* = 7).

**Table 1:** Age distribution of the patients at diagnosis

Group	Number	%
0–1 year	16	11.9
1–6 years	27	20.1
6–10 years	66	49.3
> 10 years	25	18.7
Total	134	100.0

Premature pubarche was the most frequent symptom found in the symptomatic patients with 73.5%, followed by acne 22.2%, clitoris hypertrophy 19.5%, hirsutism 14.4% and seborrhoea 10.3%. It must be borne in mind that some symptoms did not present in isolation but also in different combinations with other symptoms. The average time from the start of symptoms to diagnosis was more than 1 year. At the time of diagnosis, the mean height-SDS was 0.8 ± 1.3 (SD) and the BMI-SDS was 0.8 ± 1.2 (SD). Bone age was determined in 97/117 patients and was, on average, 9.3 ± 3.7 (SD) years. The difference between bone age and chronological age was on average 1.9 ± 1.4 (SD) years. Bone age was accelerated in 70 patients (72.2%) > 1 year.

### Laboratory diagnostics

**Fig. 1:**
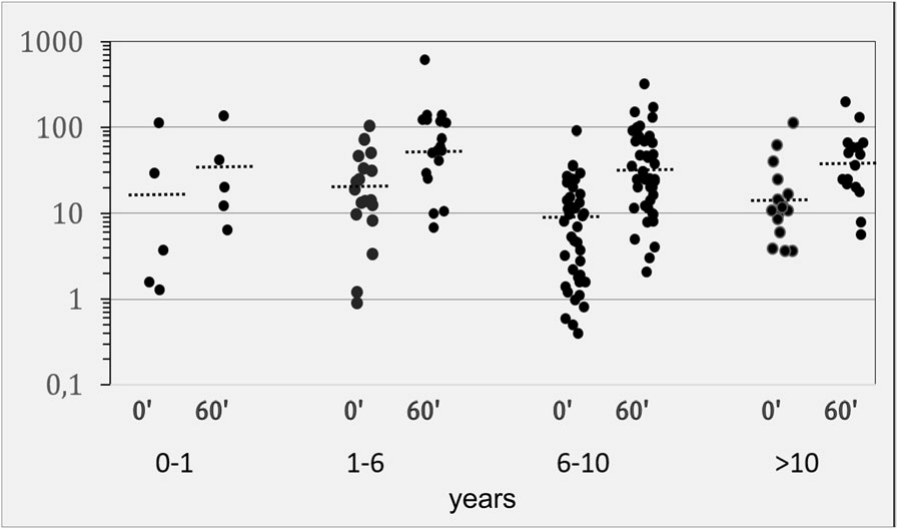
Serum concentrations of 17OHP (ng/ml; logarithmic scale) in the ACTH test (0, 60 min) in relation to the different age groups (years); 0–1 (*n* = 5), 1–6 (*n* = 18), and 6–10 (*n* = 43), > 10 (*n* = 17); black dotted line, median; conversion factor to SI, ng/ml × 3 = nmol/l

On the first visit, the mean basal 17OHP concentrations (*n* = 130) were 14.5 ± 19.1 (SD) ng/ml (range, 0.3-112). No significant differences between the different age groups were seen either for the basal or for the stimulated 17-OHP (Fig. 1). The basal 17OHP levels were < 2 ng/ml in 18 patients and > 2 ng/ml in 112 patients. In the ACTH test (*n* = 83), the 17OHP concentrations increased from an average of 18.8 ± 24.3 (SD) to 61.1 ± 79.9 (SD) ng/ml after 60 min. In 13 patients with a basal 17OHP < 2 ng/ml, the ACTH-stimulated 17OHP levels were > 10 ng/ml in 5 patients, whereas the levels did not increase in 8 patients.

Cortisol levels (ng/ml) were measured in the ACTH test in 74 patients and could be stimulated from mean 127.2 ± 57.2 (SD) to 225.9 ± 79.1 (SD). After ACTH stimulation, 20 patients had maximal cortisol levels of < 180 ng/ml (< 500 nmol/L).

### Molecular analysis

We identified 233 alleles with a mutation in the CYP21A2 gene in 126 patients. Of these, about 58% belong to the group with mild mutations and 34.7% to the severe mutations. The point mutation p.Val281Leu was found most frequently in the mild mutations (39.1%), followed by p.Pro453Ser (8.2%) and p.Pro30Leu (6.9%) mutations. A deletion of the CYP21A2 gene was most frequent among the severe mutations (11.1%), followed by the p.Ile172Asn mutation (6.9%), small gene conversions (6.0%) and the c.290-13A/C>G mutation (5.2%). There was a deletion of the pseudogene CYP21A1P on five alleles (2.1%). The mutation p.Gln318X in combination with a heterozygous duplication of the CYP21A2 gene was found on two alleles (0.9%).

Most of the patients (65.8%) were compound heterozygous with both a severe and a mild mutation, and they were assigned to group C1. In group C2, there were 21 patients, each with mild mutations (heterozygous: *n* = 11, homozygous: *n* = 10) on both alleles (16.6%). Only one mutation was found in 17 individuals (13.4%) (group C3). Patients with deletions of the pseudogene and with a duplication of the CYP21A2 gene were not included.

### Genotype-phenotype correlation

As shown in Table 2, group C1 comprised 83 patients (63 f, 20 m) with both a severe and a mild mutation of the CYPA2-gene. Of these, 71 patients (85.5%) were symptomatic. Group C2 comprised 21 patients (19 f, 2 m) with two mild mutations. In this group, all patients except one boy were symptomatic (95.2%). Group C3 encompassed the individuals (13 f, 4 m) with a heterozygous CYP21A2 gene mutation. Here, too, 94.1% (16/17) were symptomatic.

**Table 2:** Demographic and clinical characteristics of the mutation groups C1, C2 and C3; mean ± standard deviation (SD); conversion factors: 17OHP ng/ml × 3 = nmol/L, cortisol ng/ml × 27.6 = nmol/L

Groups	C1	C2	C3	*p*
Mutations	severe/mild	mild/mild	heterozygous	
Number of patients/individuals (*n*)	83	21	17	
Sex (f/m)	63/20	19/2	13/4	
Chronological age (CA) at diagnosis (years)	6.9 ± 4.3	6.2 ± 3.3	9.0 ± 5.1	ns
Symptomatic [*n* (%)]	71 (85.5)	20 (95.2)	16 (94.1)	ns
Symptoms (%)				
Premature pubarche	73.2	85.0	68.8	ns
Acne	23.9	30.0	18.8	ns
Clitoris hypertrophy*	17.2	15.7	12.5	ns
Hirsutism*	9.5	5.2	3.1	< 0.01 a
Seborrhoea	9.9	5.0	18.8	< 0.05 ^c^
Height (SDS)	0.8 ± 1.3	1.1 ± 1.3	0.2 ± 1.3	ns
Bone Age (BA) > 1 year (%)	73.3	84.2	80.0	ns
Delta BA-CA (years)	2.0 ± 1.5	1.9 ± 1.4	1.8 ± 1.0	ns
17OHP (ng/ml)	19.5 ± 22.4	9.4 ± 7.4	4.1 ± 6.6	< 0.01a^,^b
				< 0.05c
ACTH test (*n*)	46	14	13	
17OHP (ng/ml) 0 min	27.3 ± 28.8	12.7 ± 11.1	2.7 ± 2.6	< 0.01a^,^b^,^c
17OHP after ACTH	83.7 ± 97.4	58.7 ± 37.3	9.0 ± 4.2	< 0.001a^,^c
Cortisol (ng/ml) 0 min	119.4 ± 53.0	134.9 ± 56.8	112.8 ± 69.8	ns
Cortisol after ACTH	192.0 ± 62.5	218.4 ± 50.1	297.3 ± 98.7	< 0.01^a,c^

*ns* not significant

*In relation to the number of girls

^a^Significant differences between groups C1 and C3

^b^Significant differences between groups C1 and C2

^c^Significant differences between groups C2 and C3

**Fig. 2:**
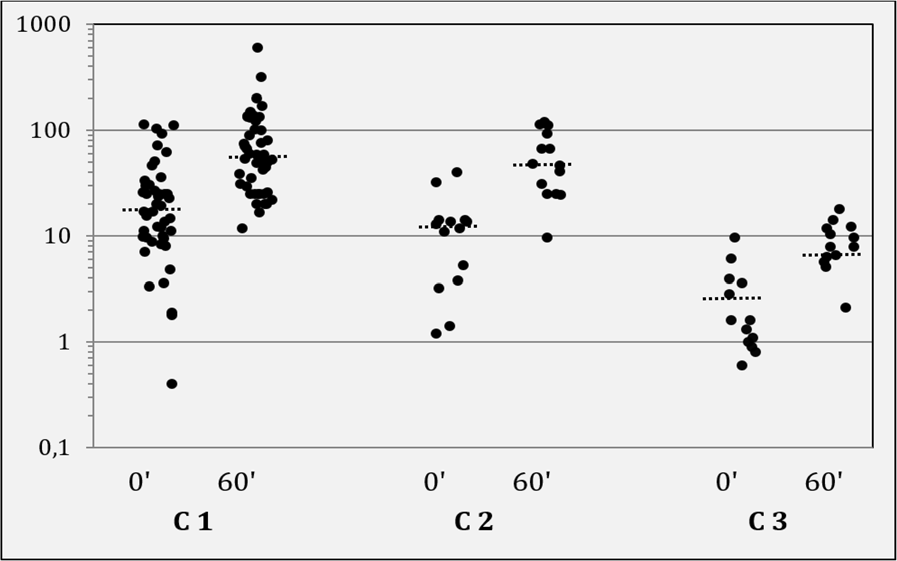
Serum concentrations of 17OHP (ng/ml; logarithmic scale) in the ACTH test (0, 60 min) in relation to the mutation groups: C1 (severe/mild; *n* = 46), C2 (mild/mild; *n* = 14), and C3 (heterozygous; *n* = 13); black dotted line, median; conversion factor to SI, ng/ml × 3 = nmol/l

Premature pubarche was the most frequent symptom in all three mutation groups. No significant phenotypical differences were found between the three mutation groups; however, marked differences were seen in the hormone levels. The basal 17-OHP concentrations in group C1 were significantly higher than in C2 (*p* < 0.001) and C3 (*p* < 0.001). The difference between C2 and C3 was also significant (*p* < 0.05). After ACTH the stimulated 17-OHP in groups C1 and C2 were significantly greater than in C3 (*p* < 0.01); no such differences were found between C2 and C3 (Fig. 2). No difference was seen in basal cortisol levels between the 3 groups. After ACTH stimulation, the maximal cortisol level in the groups C1 and C2 was significantly lower than in C3 (*p* < 0.01), while no differences were found between groups C1 and C2. Insufficient cortisol reserves (max. cortisol < 180 ng/ml) were seen in 20 patients (C1: *n* = 18; C2: *n* = 2).

## Discussion

Classic CAH occurs in two clinical forms, a form with salt wasting and a simple virilising form without aldosterone deficiency [11–13], whereas, as a rule, only symptoms of increased androgen production are seen in NC-CAH [14, 15]. The clinical symptoms vary greatly. When the symptoms are less pronounced, many patients, and particularly the boys, will be diagnosed only by chance or not at all [2, 16]. In almost all published studies, the number of male patients was markedly smaller than the number of female patients [6, 17–19]. In our cohort, the mean age of the patients at diagnosis was 7.1 years and 8.3 years in a comparable study in Turkey [19]. In most of the studies, the mean age is markedly higher, since adults were also examined. The most frequently reported clinical symptom is premature pubarche, with the details on frequency ranging from 45.3 [19] to 88.3% [18]. If the symptom is considered in relation to sex, the frequency of premature pubarche in girls and boys in our cohort was almost equal, while it was conspicuously more frequent in girls (88.5%) than in boys (28.9%) in the Greek cohort [18]. At birth, most of the girls with NC-CAH have unobtrusive external genitalia. However, clitoris hypertrophy may be noticeable when the diagnosis is made. The reported frequencies, ranging from 3.1 [19] to 13.4% [20] are lower than in our cohort with 19.5%. The signs of androgenisation increase during puberty and in adulthood [15, 21]. The numbers on hirsutism vary between 28.6 and 53% [19, 20]. We are not able to evaluate the linear growth of the children since no old growth data were available at the time of diagnosis. Bone age should also be determined in children with androgenisation. In our cohort, bone age was determined in 97 patients, and in 70 (72.2%) patients, it was > 1 year accelerated. Symptomatic children can have increased linear growth and accelerated bone age [22]; the final height may, however, be reduced [23]. Children without clinical symptoms have normal linear growth [24]. In our cohort, 12.4% and in the Greek cohort, 14.6% of patients were asymptomatic [18].

The mild mutation p.Val281Leu in Exon 7 is found most frequently on the alleles, with frequencies lying between 25 and 50% [16, 18, 19, 25]. In our cohort, this mutation was found most frequently (39%), followed by mutations p.Pro453S (Exon 10) and Pro30L (Exon 1). The most frequent severe mutation in our cohort was the deletion of the CYP21A2-gene (11.1%), whereas the Intron 2-mutation IVS2-13A/C>G was seen most frequently in the Greek cohort [18]. Most of the patients are compound heterozygous for two different mutations of the CYP21A2-gene on both alleles. In our cohort, 68.5% the patients were compound heterozygous for a severe (classic) and a mild (non-classic) mutation, whereas the percentage (severe/mild) range from 50.4% in the Greek cohort [18] to 12.6% in the Turkish cohort [19]. The number of our patients with mild mutations (16.6%) was lower than in the Turkish cohort (34%) and the portion of heterozygous patients in our study (13.4%) was also markedly lower than in the Turkish population, which was 47.8% [19]. The diagnosis was made by molecular genetic analysis in seven children during examination of family members with affected siblings at the request of the parents.

The basal 17-OHP concentration in NC-CAH patients is usually raised [14]. Higher 17OHP levels were found in the literature in adolescent girls than in children [20], although we were unable to determine any age differences. Significantly, higher basal 17-OHP levels were found in patients with a compound heterozygous mutation (severe/mild) than in patients with two mild mutations [18, 21, 25, 26]. In our cohort, the patients in group 1 (compound heterozygous mutation: severe/mild) also had higher basal values, and in the ACTH test, they also had higher stimulated 17OHP-values than the patients with two mild mutations (group 2), but the difference was not statistically significant. Basal 17OHP levels < 2 ng/ml should rule out NC-CAH [13, 27, 28]. This proposition is not shared by all working groups [18, 21, 29]. In our cohort, 4 (3.8%) out of 101 patients with a molecular-genetically confirmed NC-CAH (groups 1 and 2) and 6 (2.2%) out of 280 patients in the Greek cohort had a basal 17OHP level of < 2 ng/ml [18].

The ACTH stimulation test is regarded as the gold standard in laboratory diagnostics—also in order to differentiate from other non-classic CAH forms with 11β-hydroxylase- or 3β-hydroxysteroid-dehydrogenase deficiency [2]. In our cohort, the test was performed in only 81 (61.9%) out of 134 patients and in 220 (85.2%) out of 258 patients in the Turkish study. 17OHP levels from > 10 ng/ml after ACTH stimulation are considered a diagnosis for NC-CAH [13, 21, 30, 31]. However, in an assessment of women with clinical hyperandrogenism, only 24% of them with a stimulated 17OHP > 10 ng/ml had a molecular-genetically confirmed NC-CAH [32]. In our cohort, all patients with NC-CAH had ACTH-stimulated 17OHP values > 10 ng/ml, whereas in the Turkish cohort the percentage was 91.8% [19]. Heterozygous gene carriers can also be identified with the ACTH test, although here there is a greater overlap with the healthy population [31, 33]. Stimulated 17-OHP levels between 2 and 10 ng/ml have been reported in heterozygous gene carriers [34]. Our results show that a cut-off level of 17OHP > 10 ng/ml in the ACTH test does not definitely distinguish heterozygous gene carriers from patients. Of the molecular-genetically confirmed heterozygous individuals in our cohort (group C3), five had an ACTH-stimulated 17OHP level > 10 ng/ml and 8 patients had levels < 10 ng/ml.

Since most patients have normal cortisol levels (basal and 60 min after ACTH) and thus react adequately to stress [35–37], measuring cortisol in the ACTH test is not considered necessary and is not critically examined. In our cohort, a standard ACTH test was performed in 83 patients and cortisol was measured in 73 patients. ACTH-stimulated cortisol values < 180 ng/ml (< 500 nmol/l) are generally considered to be pathological and are regarded as diminished cortisol reserves. A French working group was able to show that 60% of children with NC-CAH had diminished cortisol reserves [38]. In our cohort, the proportion was 27.4%, and in other studies, it is between 15.4 and 21.5% [19, 39]. To date, no generally accepted guidelines for therapy in stress situations have been developed for patients with NC-CAH [40]. Patients with diminished cortisol reserves can only be detected with an ACTH test and receive adequate therapy in stress situations [38].

No significant phenotypical differences were found in a comparison of the three mutation groups. It is remarkable that heterozygous individuals have the same clinical symptoms as the patients in groups C1 and C2. It is a known fact that heterozygous gene carriers have symptoms of hyperandrogenism [41]. Women with hyperandrogenism are more frequently identified as heterozygous gene carriers for a non-classic CYP21A2 mutation than in the general population [42, 43], but to date, there is no precise explanation for the symptoms described in the heterozygous patients [44].

## Conclusion

The clinical symptoms in patients with non-classic CAH are variable and sometimes only very mild. The symptoms in the molecular-genetically heterozygous patients are no different from those in the patients with compound heterozygous (severe/mild) or homozygous (mild/mild) mutations. We speculate that many patients, in particular males, have not yet been diagnosed. This is evidenced by the fact that the number of patients who are being treated in the centres involved is lower by a factor of about 5 than the number of patients with classic CAH with 21-hydroxylase defect. The clinical and laboratory diagnostics performed when NC-CAH is suspected vary in the different centres. The variability in the laboratory findings demonstrates that a definitive diagnosis can only be confirmed molecular-genetically. It is therefore imperative that a consensus is reached among paediatric endocrinologists in relation to diagnosis of children with NC-CAH.

## Data Availability

Not applicable.

## Abbreviations

CYP21A2: Cytochrome P450 Family 21 Subfamily A Member 2
CAH: Congenital adrenal hyperplasia
NC-CAH: Nonclassical congenital adrenal hyperplasia
MLPA: Multiplex ligation-dependent probe amplification
BMI: Body mass index
SDS: Standard deviation score
17-OHP: 17-Hydroxyprogesterone
CA: chronological age
BA: bone age
ACTH: Adrenocorticotropic hormone
ANOVA: Analysis of variance
